# *In vitro* and *in vivo* assessment of inhibitory effect of stevioside on pro-inflammatory cytokines

**Published:** 2017

**Authors:** Jatuporn Noosud, Narissara Lailerd, Autchara Kayan, Chaiwat Boonkaewwan

**Affiliations:** 1 *Department of Companion Animal Clinical Science, Faculty of Veterinary Medicine, Kasetsart University, Bangkok, Thailand*; 2 *Department of Physiology, Faculty of Medicine, Chiang Mai University, Chiang Mai, Thailand*; 3 *Department of Animal Science, Faculty of Agriculture, Kasetsart University, Bangkok, Thailand*

## Abstract

**Objective:**

Stevioside is a natural non-caloric sweetener which has been reported to have anti-inflammatory activity. The aim of the present study was to examine *in vitro *and* in vivo* effects of stevioside on rats plasma levels of tumor necrosis factor- α (TNF-α), interleukin-1β (IL-1β), TNF-α and IL-1β release from lipopolysaccharide(LPS)-stimulated rat peripheral blood mononuclear cells (PBMCs).

**Materials and Methods:**

Male wistar rats weighing between 170-220 g were given stevioside (0, 500 and 1000 mg/kg BW/day) for 6 weeks. Mononuclear cells were separated from peripheral blood samples. TNF-α and IL-1β levels in plasma and the release of TNF-α and IL-1β from PBMCs were determined using rat enzyme-linked immunosorbent assay (ELISA) kits.

**Results:**

Plasma levels of TNF-α and IL-1β were found to be non-detectable in control and groups treated with 500 and 1000 mg/kg of stevioside. Regarding TNF-α release from LPS-stimulated PBMCs, rats that were orally fed with 500 and 1000 mg/kg of stevioside were significantly different (p<0.05) from those in LPS-treated control group (186.8+18.6 and 151.4 + 15.4 vs 248.6+21.4 pg/ml). Additionally, IL-1β levels in rats treated with 500 and 1000 mg/kg of stevioside were significantly different (p<0.05) from those in LPS-treated control group (220.0+12.1 and 158.1 + 22.6 vs 294.4+16.1 pg/ml).

**Conclusion:**

Consumption of stevioside has an inhibitory effect on the release of TNF-α and IL-1β from LPS-stimulated PBMCs in rats.

## Introduction

Stevioside (SVS), a natural non-caloric sweetener isolated from *Stevia rebaudiana *Bertoni, is approximately 300 times sweeter than sucrose (Hanson and Oliverira, 1993 ▶). It is a diterpeniccarboxylic alcohol with three glucose molecules, and has a molecular weight of 804.9. It does not appear to be taken up across the intestinal mucosa, when given orally (Koyama et al., 2003 ▶). The major metabolite of stevioside is the alcoholic form, steviol, which has a molecular weight of 318.44 (Mosetting, 1995 ▶). Stevioside is not degraded into steviol by any of the mammalian enzymes including those in human digestive tract (Koyama et al., 2003 ▶). Only the bacteria from the caecum or colon are able to degrade stevioside into steviol (Geuns et al., 2007 ▶). Stevioside has been used as a sugar substitute in several countries such as Japan, Korea, Brazil, the United States and Thailand (Chatsudthipong and Muanprasat, 2009 ▶). Moreover, stevioside has been shown to exert beneficial effects including anti-hyperglycemic (Jeppesen et al., 2006 ▶), anti-hypertension (Lee et al., 2001 ▶), anti-inflammation (Boonkaewwan et al., 2006 ▶; Boonkaewwan et al., 2008 ▶; Boonkaewwan and Burodom, 2013 ▶), and anti-diarrhea (Pariwat et al., 2008 ▶) activities in human.

Gram-negative bacterial cell wall, in particular the lipopolysaccharide (LPS), can stimulate monocytes and macrophages immune cells to release inflammatory cytokines. Among them, the pro-inflammatory cytokines, TNF-α and interleukin (IL)-1β are important inflammatory mediators reported to be involved in the development of a number of inflammatory diseases (Freeman and Natanson, 2000 ▶). Alteration of production of TNF-α and IL-1β can be employed as criteria for evaluation of anti-inflammatory effects of the natural products. 

Stevioside and steviol have shown to have anti-inflammatory activity, *in vitro*. However, determination of stevioside and steviol concentrations *in vivo* would be complicated by several factors such as dilution and bacterial flora; therefore, it was interesting to study the effect of oral ingestion of stevioside on the immunological functions. This study aimed to examine *in vivo* effect of stevioside on plasma levels of TNF-α and IL-1β and TNF-α and IL-1β release from rats PBMCs that were stimulated with LPS.

## Materials and Methods

**Preparation of stevioside**

**Figure 1:**
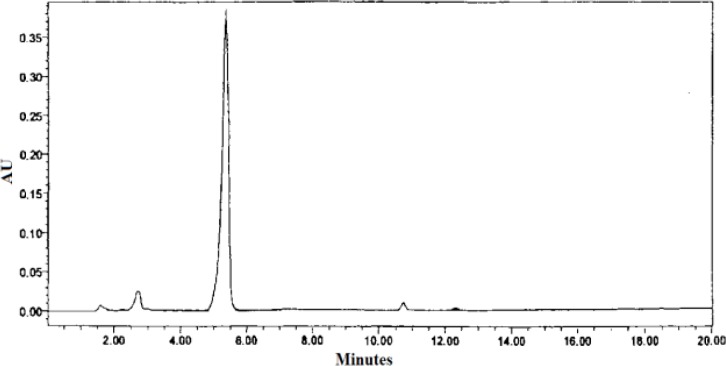
The purity of stevioside

Crude stevioside was supplied by Thai Pharmacognosy Research Laboratory, Chaing Mai. Stevioside (approx.96-98% purity; Figure 1) was extracted and purified from dried *S. rebaudiana* leaves as described by Adduci et al (1987) ▶. Purity of stevioside was determined using High-performance liquid chromatography conducted with a Waters model 510 liquid chromatograph (Waters, Millipore Corp., Milford, MA).

**Experimental animals**

Male Wistar rats weighing 170-220 g were obtained from the National Animal Center (NLAC), Mahidol University, Nakornpathom, Thailand. Animals were individually kept in stainless cages in an animal room at 24-25 ºC, with relative humidity of 65% and a 12 hr:12 hr dark-light cycle. Animals were fed with regular rat chow (C.P. Food, Pokphan animal Feed Co. Ltd., Bangkok) and water, *ad libitum*. The animals were acclimatized for one week before being used in this experiment. Rats were randomly divided into the following 3 groups of 6 rats. Group I: rats were given water via oral gavage at the same volume as that given to the treated animals. Groups II and III: Rats were given stevioside (500 and 1000 mg/kg BW/ day) for 6 weeks.

**Blood and plasma collection**

After the end of treatment, blood (4 ml) was collected and thoroughly mixed with EDTA. Then, 1 ml of blood was centrifuged at 10000 g at 25 º C for 10 min to separate the plasma. Plasma was collected and stored in appropriate tubes at -80 ºC until the time of determination of TNF-α and IL-1β levels. Also, 3 ml of blood was used for PBMCs isolation.

**Isolation of peripheral blood mononuclear cells (PBMCs)**

Mononuclear cells were separated from peripheral blood samples according to the method described by Boyum (1968) ▶. Briefly, 3 ml of blood was gently layered over 2 ml of lymphoprep solution and centrifuged at 400 g for 15 min. The white band of mononuclear cells was collected and washed 3 times with RPMI 1640 culture medium by centrifugation at 800 g for 5 min. PBMCs were resuspended in complete RPMI 1640 culture medium (RPMI 1640 medium containing 25 mM HEPES, 2 mM L-glutamine, 10% heat-inactivated fetal calf serum, penicillin (100 U/ml) and streptomycin (100 µg/ml)) and adjusted to 2 × 10^6^ cells/ml. Cell viability assay was done using trypan blue dye exclusion technique. 

**Determination of TNF-α and IL-1β production**

PBMCs (2 × 10^6^ cells/ml) were separated from each rat and incubated with or without LPS (1 µg/ml) for 24 hr in a humidified atmosphere of 5% CO2 at 37 ºC. Supernatant fluids were collected and stored at -80º C until measurement of TNF-α and IL-1β. TNF-α and IL-1β levels in supernatant were determined using commercial enzyme-linked immunosorbent assay (ELISA) kits (R&D systems, Minneapolis, MN, USA) according to manufacturer’s instructions.

**Statistical analysis**

Data from at least three individual experiments were analyzed and presented as mean ± SEM. Statistical significance was determined using one-way ANOVA and student Newman-Keuls. A p<0.05 was considered as statistically significant.

## Results

**Effect of stevioside on cell viability**

To examine the effect of stevioside ingestion on PBMCs viability, we used trypan blue dye exclusion technique. Results showed that oral ingestion of stevioside (500 and 1000 mg/kg BW/day) for 6 weeks had no toxicity in PBMCs. The cell viability in non-treated control and stevioside treated groups were similar (nearly 100% viability) (data not shown). 

**Plasma levels of TNF-α and IL-1β**

At the end of treatment, plasma levels of TNF-α and IL-1β were determined. Results demonstrated that TNF-α and IL-1β were non-detectable in rats plasma in control and groups treated with 500 and 1000 mg/kg BW of stevioside.

**TNF-α release from LPS-stimulated PBMCs**

**Figure 2:**
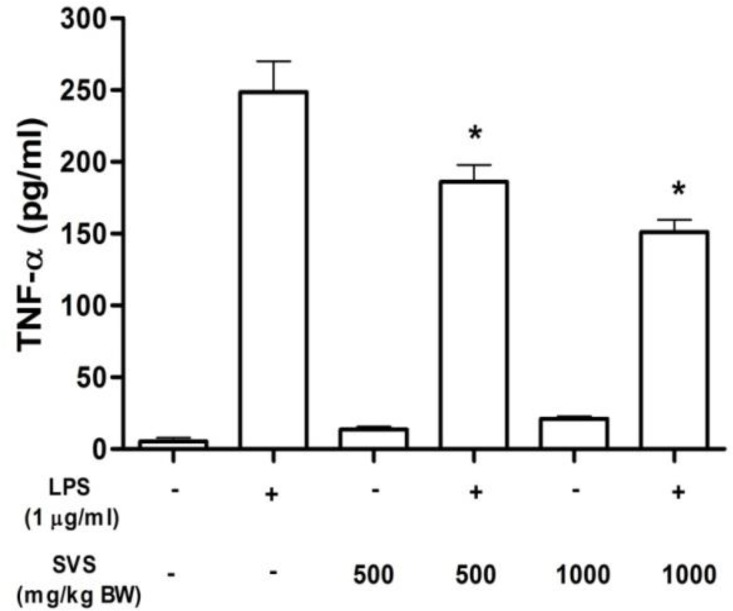
Effect of orally given stevioside on TNF-α release in rats**. **PBMC (2 × 10^6^ cells) from each group was incubated for 24 hr in the presence or absence of LPS (1 µg/ml). *Statistically significant difference in TNF-α release (p<0.05) as compared to LPS-treated in control group (n=5

TNF-α level in non-LPS-stimulated PBMCs from non-treated control and groups treated with 500 and 1000 mg/kg BW of stevioside were 5.4±4.4, 13.7±3.1 and 20.7±3.2 pg/ml, respectively. TNF-α release from rats PBMC stimulated with LPS were compared between control and stevioside-treated groups. Level of TNF-α in LPS-stimulated PBMCs in control group was 248.6+21.4 pg/ml. TNF-α level in LPS-stimulated PBMC in groups treated with 500 and 1000 mg/kg BW of stevioside were 186.8±18.6 and 151.4±15.4 pg/ml, respectively (Figure 2). This result demonstrated that TNF-α level in LPS-stimulated PBMC in rats orally fed with 500 and 1000 mg/kg BW of stevioside was significantly decreased as compared to the control group (p<0.05).

**IL-1β release from LPS-stimulated PBMCs**

**Figure 3:**
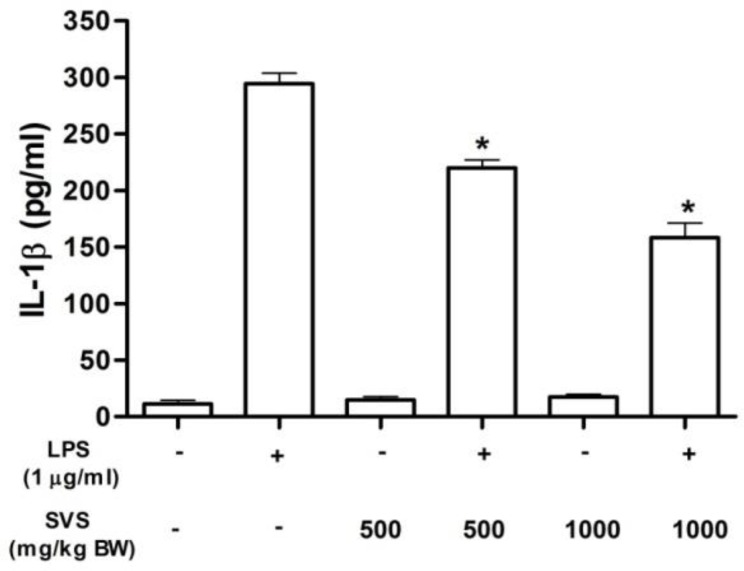
Effect of orally given stevioside on IL-1β release in rats. PBMCs (2 × 10^6^ cells) from each group was incubated for 24 hr in the presence or absence of LPS (1 µg/ml). *Statistically significant difference in IL-1β release (p<0.05), as compared to LPS-treated in the control group (n=5

IL-1β level in non-LPS-stimulated PBMCs from non-treated control and groups treated with 500 and 1000 mg/kg BW of stevioside were 11.2±5.8, 14.7±5.1 and 17.2±4.6 pg/ml, respectively. IL-1β release from LPS-stimulated PBMCs in control group was 294.4+16.1 pg/ml. IL-1β level in LPS-stimulated PBMC in rats orally fed with 500 and 1000 mg/kg BW of stevioside were 220.0±12.1 and 158.1±22.6 pg/ml, respectively (Figure 3). Results indicated that IL-1β level in LPS-stimulated PBMC in rats orally fed with 500 and 1000 mg/kg BW of stevioside were significantly decreased as compare to non-treated control (p<0.05***).***

## Discussion

The metabolism of stevioside in human volunteers demonstrated that all the stevioside reaching the colon was degraded by micro-organisms into steviol, the only metabolite found in feces. In blood plasma, no stevioside, no free steviol or other free steviol metabolites were found (Guens et al., 2007 ▶). It was interesting to study the effect of oral administration of stevioside on the immunological function. The present study was undertaken to examine effect of stevioside on plasma levels of TNF-α and IL-1β and TNF-α and IL-1β release from rat PBMCs that were stimulated with LPS, *in vivo*. 

We first examined the effect of stevioside ingestion on PBMCs viability by using trypan blue dye exclusion test before studying the cytokine release. PBMCs are blood cells having round nucleus, such as a lymphocyte or a monocyte. These blood cells are critical components of the immune system. The most straightforward method for determining viable cell number is a direct counting of cells by a hemocytometer. It is more helpful to stain the cells with a dye such as trypan blue, since viability of cells could be measured by the ability of live cells with uncompromised membrane integrity to exclude the dye. Our study showed that oral administration of stevioside had no toxicity in rat PBMCs. This result is similar an *in vitro* study which reported that stevioside had no cytotoxic effect on THP-1 (human monocytic cell) and Caco-2 (colon cancer cell) cells line (Boonkaewwan et al., 2006 ▶ and Boonkaewwan and Burodom, 2013 ▶).

TNF-α and IL-1β are biologically active peptides produced by monocytes, and their production is induced by endotoxin and other stimuli. These cytokines are important for host survival from infection, while their overproduction has deleterious effects. Therefore, synthesis of pro-inflammatory cytokines must be tightly controlled (Morikawa et al., 1996 ▶). Stevioside (500 and 1000 mg/kg BW/day) did not have any effect on plasma levels of TNF-α and IL-1β in rats. In general, TNF-α and IL-1β are not usually detectable in healthy individuals and the elevation of plasma and tissue levels of these cytokines is mostly seen in inflammatory and infectious conditions (Robak et al., 1998 ▶ and Rabinovitch and Suarex-Pinzon, 2003 ▶). On the other hand, an * in vitro* study in THP-1 cell demonstrated that 1 mM of stevioside induced TNF-α release, which may be partially mediated via TLR4. The three glucose molecules that are present only in stevioside may play a crucial role in stevioside interaction with THP-1 cells (Boonkaewwan et al., 2008 ▶). 

The Joint FAO/WHO Expert Committee on Food Additives (JECFA, 2008 ▶) established the permanent accepted daily intake (ADI) for stevioside at 0-11 mg/kg/day based on no-observable genotoxic effect in human and experimental animals. The present study used high concentrations of stevioside because our laboratory previously found that these concentrations (500 and 1000 mg/kg BW/day) markedly decreased hyperglycemia in streptozotocin-induced diabetic rats without alteration of the basal plasma insulin levels, and also improved some clinical signs of diabetes. The present study demonstrated that PBMCs isolated from rats treated with stevioside (500 and 1000 mg/kg BW/day) showed a reduction in TNF-α and IL-1β release from LPS-stimulated PBMCs (Figures 2 and 3). Similar to previous studies, stevioside attenuated LPS-induced pro-inflammatory cytokine release in THP-1, T84 and Caco-2 cells (Boonkaewwan et al., 2006 ▶; Boonkaewwan et al., 2008 ▶; Boonkaewwan and Burodom, 2013 ▶). These information support an inhibitory effect of stevioside on the release of TNF-α and IL-1β. Since the metabolism of stevioside in human volunteers demonstrated that only steviol glucuronide was found in blood (Guens et al., 2007 ▶), therefore, it is possible that the inhibitory effect of oral administration of stevioside on the responsiveness of LPS-stimulated PBMCs could be possibly from the action steviolglucoronide. However, further studies should be conducted to investigate the action of steviolglucoronide which is the only metabolite that was found in plasma.

Consumption of stevioside has an inhibitory effect on the release of TNF-α and IL-1β from LPS-stimulated PBMCs. This would provide beneficial support for further investigation on its therapeutic application in inflammatory and infectious condition.
